# Time-varying overdispersion of SARS-CoV-2 transmission during the periods when different variants of concern were circulating in Japan

**DOI:** 10.1038/s41598-023-38007-x

**Published:** 2023-08-14

**Authors:** Yura K. Ko, Yuki Furuse, Kanako Otani, Masato Yamauchi, Kota Ninomiya, Mayuko Saito, Takeaki Imamura, Alex R. Cook, Tadayuki Ahiko, Shunji Fujii, Yoshiharu Mori, Emiko Suzuki, Keiko Yamada, Yoshikazu Ashino, Hidetoshi Yamashita, Yuichi Kato, Katsumi Mizuta, Motoi Suzuki, Hitoshi Oshitani

**Affiliations:** 1 Department of Virology, Tohoku University Graduate School of Medicine, 2-1 Seiryo-Machi, Aoba-Ku, Sendai, Miyagi 980-8575 Japan; 2 Nagasaki University Graduate School of Biomedical Sciences, Nagasaki, Japan; 3 Center for Surveillance, Immunization, and Epidemiologic Research, National Institute of Infectious Diseases, Tokyo, Japan; 4 Japan International Cooperation Agency, Tokyo, Japan; 5 Graduate School of Pharmaceutical Sciences, The University of Tokyo, Tokyo, Japan; 6 Saw Swee Hock School of Public Health, National University of Singapore and National University Health System, Singapore, Singapore; 7 Division of Health and Welfare Planning, Yamagata Prefectural Government, Yamagata, Japan; 8 Murayama Public Health Center, Yamagata, Japan; 9 Mogami Public Health Center, Yamagata, Japan; 10 Okitama Public Health Center, Yamagata, Japan; 11 Shonai Public Health Center, Yamagata, Japan; 12 Yamagata City Institute of Public Health, Yamagata, Japan; 13 Yamagata Prefectural Institute of Public Health, Yamagata, Japan

**Keywords:** Infectious diseases, Public health

## Abstract

Japan has implemented a cluster-based approach for coronavirus disease 2019 (COVID-19) from the pandemic’s beginning based on the transmission heterogeneity (overdispersion) of severe acute respiratory coronavirus 2 (SARS-CoV-2). However, studies analyzing overdispersion of transmission among new variants of concerns (VOCs), especially for Omicron, were limited. Thus, we aimed to clarify how the transmission heterogeneity has changed with the emergence of VOCs (Alpha, Delta, and Omicron) using detailed contact tracing data in Yamagata Prefecture, Japan. We estimated the time-varying dispersion parameter (${k}_{t}$) by fitting a negative binomial distribution for each transmission generation. Our results showed that even after the emergence of VOCs, there was transmission heterogeneity of SARS-CoV-2, with changes in ${k}_{t}$ during each wave. Continuous monitoring of transmission dynamics is vital for implementing appropriate measures. However, a feasible and sustainable epidemiological analysis system should be established to make this possible.

## Introduction

Overdispersion is a well-known characteristic of severe acute respiratory coronavirus 2 (SARS-CoV-2) transmission^1,2^. While many infected individuals do not transmit to anyone, the chain of transmission can only be maintained through superspreading events (SSEs), in which a small number of cases generate many secondary cases. The degree of overdispersion is generally expressed by the dispersion parameter ($k$) when the distribution of the number of secondary cases is fitted to a negative binomial distribution, with a small $k$ (< 1) indicating overdispersion^3^. Theoretically, preventing SSEs can significantly reduce the transmission of SARS-CoV-2^4–6^.

Based on this characteristic, Japan has implemented cluster-based approaches that focus on identifying and preventing SSEs to minimize the impact of coronavirus disease 2019 (COVID-19) since the beginning of the pandemic^7^. Japan also identified the common environmental risk factors for SSEs, which are now known as “Three Cs”: (1) closed spaces with poor ventilation, (2) crowded spaces with many people, and (3) close contact settings. These measures were based on epidemiological investigations before the emergence of variants of concern (VOCs)^8^. Transmissibility has been increasing for each VOC, and a substantially higher secondary household attack rate has been reported for the Omicron variant^9,10^. Very few studies have shown the extent of overdispersion after the emergence of the VOCs^11–14^. There are some discussions on whether overdispersion is maintained with the Omicron variant^15^. However, only two studies have reported overdispersion of the Omicron variant as of July 2022^16,17^. Moreover, there have been no reports analyzing the degree of overdispersion of VOCs, such as Alpha, Delta, and Omicron in the same area over time.

One of the reasons for the limited reports of overdispersion, especially after the emergence of the Omicron variant, is that extensive contact tracing, including backward contact tracing, has been maintained in a few polities, almost exclusively in Asia^18,19^, and such contact tracing is no longer conducted even in these countries and areas with the rapid increase in confirmed cases since the emergence of the Omicron variant. Yamagata Prefecture is in the northern part of Japan and has a population of 1.08 million with a population density of 370 inhabitants per square kilometer. The cumulative incidence of COVID-19 per population is relatively low compared with other prefectures in Japan^20^; therefore, it has been possible to continue extensive contact tracing until the early stage of the Omicron dominant wave. In addition, local governments have made epidemiological information from these contact tracing efforts available to the public on their websites^21^.

In this study, using the line list of cases generated from the data available on the websites^22^, we conducted the analysis aiming to clarify how the degree of transmission heterogeneity has changed with the emergence of VOCs (Alpha, Delta, and Omicron). Traditionally, $k$ has been regarded as a fixed characteristic for each pathogen; however, Adam et al. recently demonstrated the importance of estimating the time-varying dispersion parameter ${(k}_{t}$)^23^. To analyze the degree of transmission heterogeneity at multiple time points more closely, we estimated the ${k}_{t}$ in addition to the average values of $k$ within time windows.

## Results

**Figure 1:**
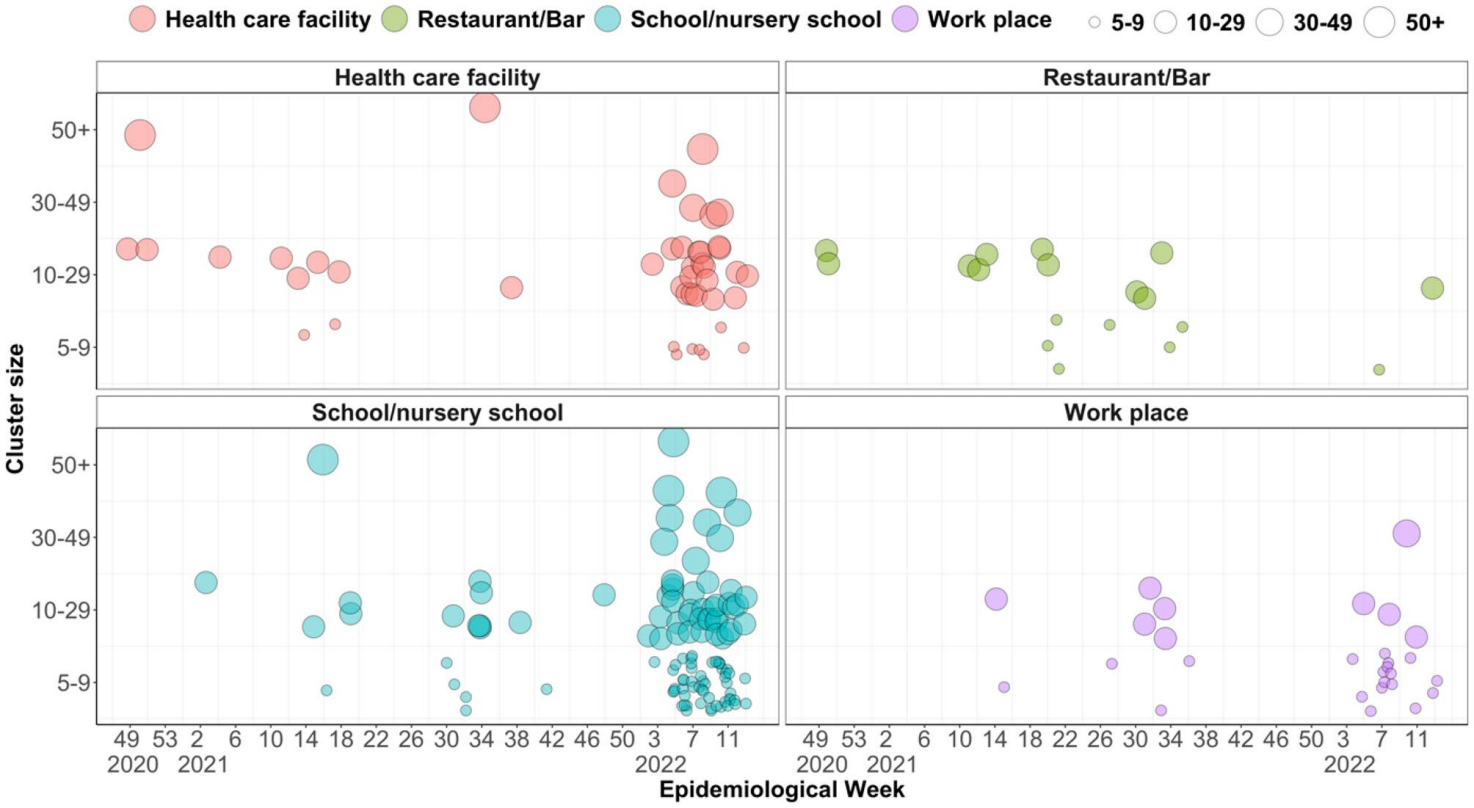
Number of clusters reported by epidemiological week, cluster type, and cluster size (the number of persons infected, indicated by circle size). “Cluster” is defined as with at least five confirmed cases within a common event or venue.

**Figure 2:**
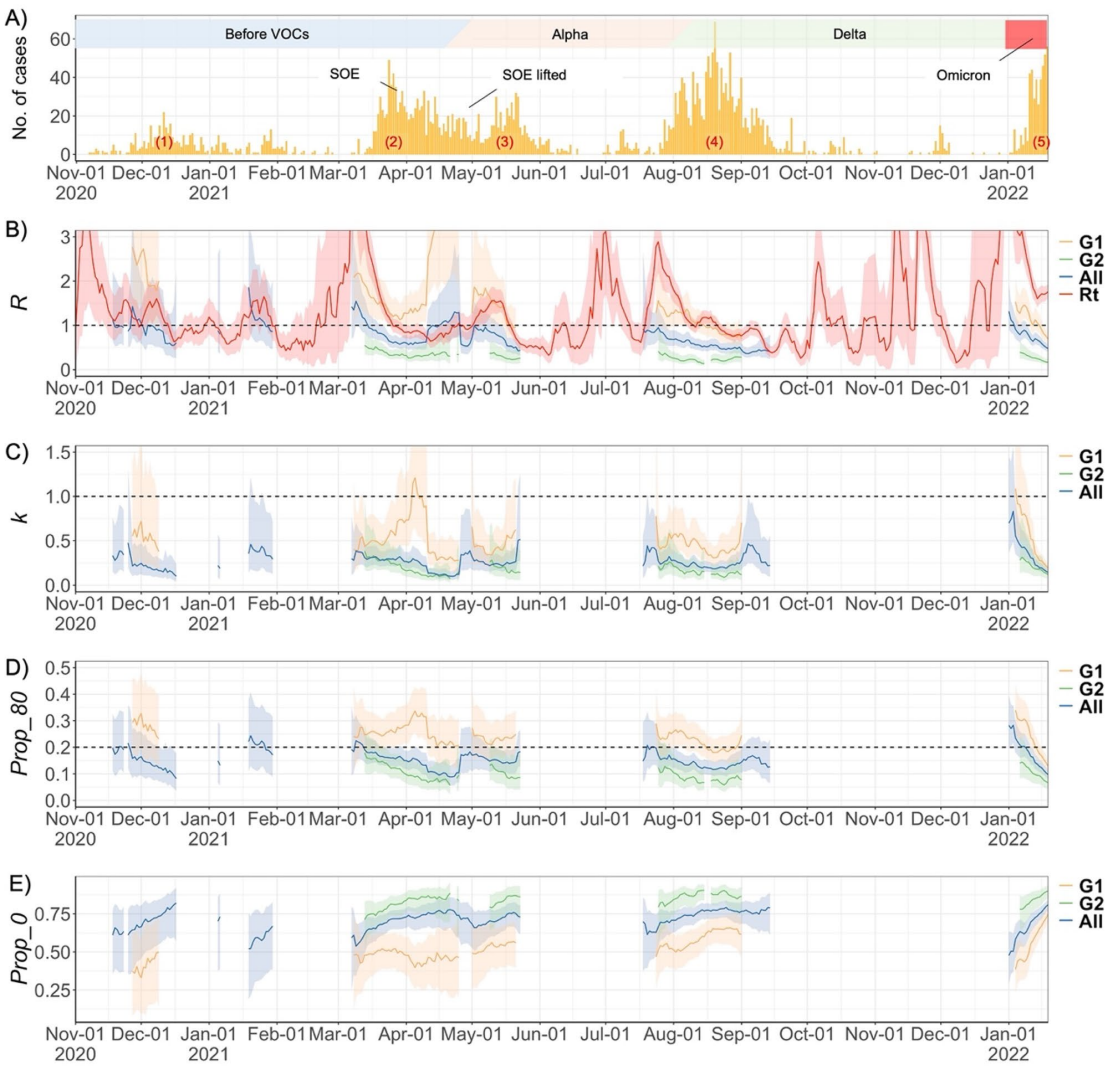
(**A**) Epidemic curve (based on confirmed date) with colored boxes indicating dominant variants at each time period, (**B**) Effective reproduction number (${R}_{t}$, or *R* on the chart), red line was estimated from the daily number of cases, (**C**) time-varying dispersion parameter (${k}_{t}$, *k* on the chart), (**D**) the proportion of cases infecting 80% (${P}_{80}$, *Prop_80* on the chart), and (**E**) the proportion of cases who did not spread to anyone (${P}_{0}$, *Prop_0* on the chart) for each generation of transmission in Yamagata, Japan between 2020 November 1 and 2022 January 19. In Panel (**A**), (1) ~ (5) indicates each wave. In Panel (**B**–**E**), the shaded areas show the 95% CrI. SOE: State of emergency, G1: first-generation, G2+: second-generation or later, All: All generation.

Yamagata Prefecture reported 211 clusters, which are defined to be a group of cases that involve five or more confirmed cases in a common event or in the same setting except for household, between November 2020 and March 2022. Until the third quarter of 2021, many clusters associated with restaurants and bars were reported, whereas there was a significant increase in the number of clusters associated with healthcare facilities and schools/nursery schools after February 2022 (Fig. 1). From the line list of all confirmed cases in Yamagata Prefecture, we obtained the data reported from November 2020 to January 2022. The daily proportion of G2 + cases (with an identified source of infection) was greater than 50% throughout the study period, except in late January 2022 (Supplementary Fig. 1A). Thus, to maintain the data quality, we limited the study period of the main analysis to January 19, 2022. Among those 3,958 cases in the study period, 1,330 cases (33.6%) were considered first-generation (G1) of transmission, and 2,628 cases (66.4%) were second-generation or later (G2+). From January 1 to January 19, 2022, when we regarded most cases as Omicron cases, 40.0% were considered G1 (172/430), and 60.0% were G2+ (258/430). In total, 42.8% of the identified sources of infection were within the same household, although short-term variations were observed (Supplementary Fig. 1B). Five epidemic waves were observed in Yamagata Prefecture during the study period (Fig. 2A): first wave: December 2020 to January 2021; second wave: March to April 2021; third wave: May 2021; fourth wave: August to September 2021; and fifth wave: January 2022.

The trend of ${R}_{t}$ estimated from the daily number of confirmed cases and ${R}_{t}$ estimated from the transmission pairs were generally consistent, especially for G1. The ${R}_{t}$ of cases of G2 + was lower than that of G1 throughout the study period (Fig. 2B). The time-varying dispersion parameter (${k}_{t}$) in G1 cases was generally below one; however, it temporarily exceeded one in early April 2021 and January 2022, followed by a sharp decrease (Fig. 2C). The ${k}_{t}$ value in G2+ was consistently lower than that in G1, and the proportion of cases responsible for 80% of transmission (${P}_{80}$) showed a similar trend, generally ranging between 0.05 and 0.35 in G1 and between 0.05 and 0.20 in G2+ (Fig. 2D). The proportion of cases who did not generate any secondary cases (${P}_{0}$) ranged from 33.0% to 74.8% for G1 and 71.2% to 90.4% for G2+ with short-term fluctuation (Fig. 2E).

**Figure 3:**
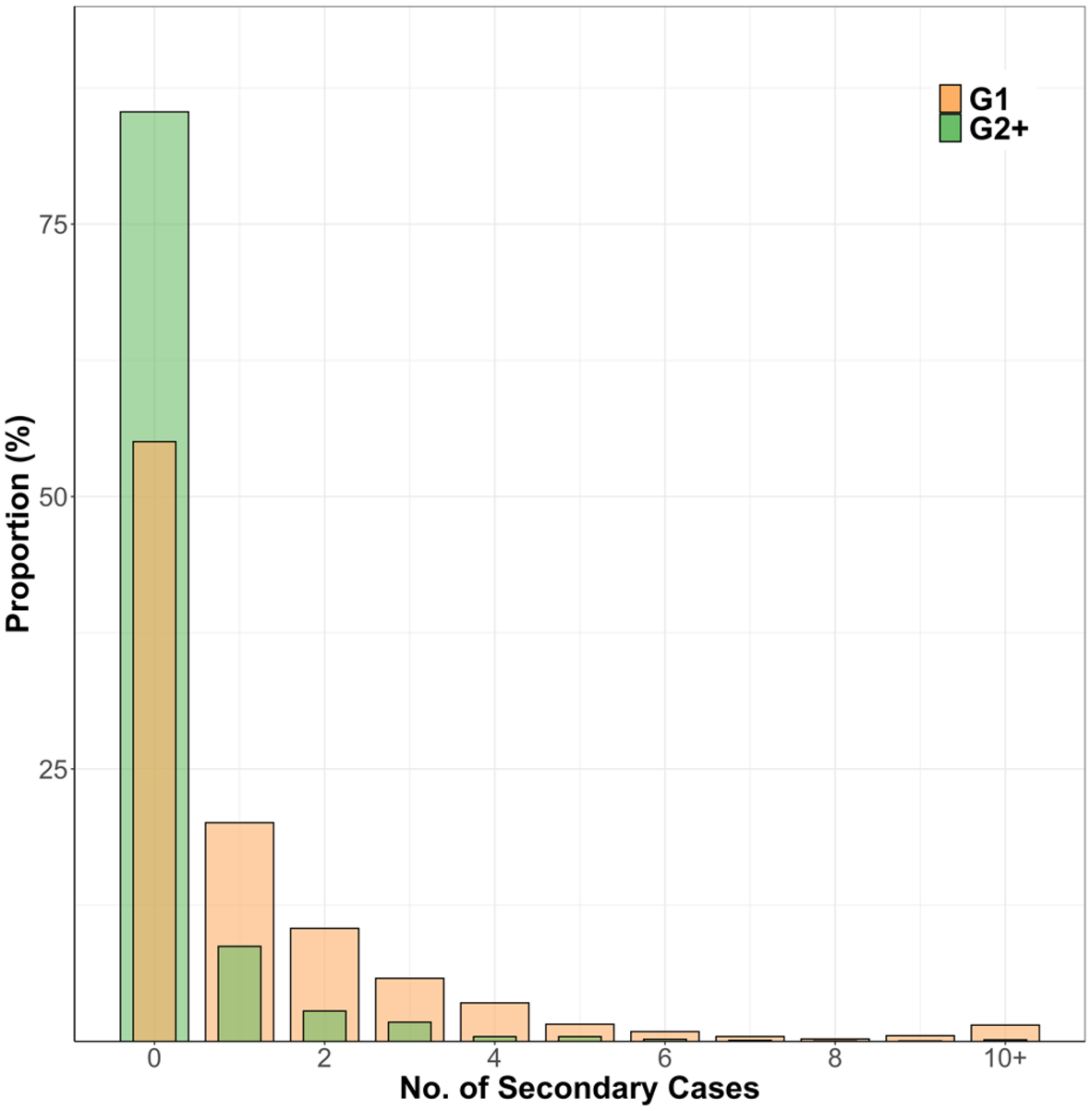
The observed offspring distribution of the number of secondary cases from G1 and G2+ cases in Yamagata, Japan. G1: first-generation, G2+: second-generation or later.

The overall dispersion parameter (*k*) for the entire study period was 0.43, 0.15, and 0.23 for G1, G2+, and all generations, respectively (Table 1). In G1, we observed 20 large clusters that had more than ten secondary cases from a single primary case, whereas only three clusters were observed in G2+ (Fig. 3). G1 had a higher ${P}_{80}$ than G2+. More than half (56%) of G1 cases did not generate any secondary cases, as did 85% of those in later generations (G2+, Table 1). The sensitivity analysis showed the same trends and results even after excluding cases associated with healthcare and other facilities (Supplementary Figs. S2, S3, and Table S1).

**Table 1:** Estimated time-fixed reproduction number ($R$), dispersion parameter ($k$), the proportion of cases responsible for 80% of the transmission events (${P}_{80}$), and the proportion of cases who did not spread to anyone (${P}_{0}$) by the generation of transmission.

Generation	*R*	*k*	*P_80*	*P_0*
G1	1.21 (1.10–1.34)	0.43 (0.38–0.50)	0.23 (0.21–0.25)	0.56 (0.52–0.60)
G2 +	0.28 (0.24–0.32)	0.15 (0.13–0.18)	0.09 (0.08–0.11)	0.85 (0.83–0.87)
All	0.65 (0.60–0.70)	0.23 (0.21–0.25)	0.14 (0.13–0.15)	0.74 (0.71–0.76)

## Discussion

This study showed, for the first time, the time-varying transmission heterogeneity for the periods in which different variants were circulating in the same area of Japan. The first two waves were mainly caused by several lineages with D614G mutation. The third wave was predominantly Alpha, the fourth wave Delta, and the fifth wave Omicron BA.1^24,25^. Our results showed that even after the emergence of VOCs, there was transmission heterogeneity of SARS-CoV-2, suggesting that preventing SSEs, for instance by reducing random contacts, can still be effective, even with VOCs^4^. However, it should be noted that the Omicron period of the present data included only the early period of the epidemic. Future studies are warranted to assess the extent of overdispersion by Omicron variants in Japan. In addition, it is also important to note that our results do not indicate that VOCs did not alter the degree of transmission heterogeneity. The degree of transmission heterogeneity depends on various factors, including the intensity of non-pharmaceutical interventions^23^, behavioral changes in people, COVID-19 vaccination^17^, and the previous infection history^26^. The combination of these factors and the emergence of VOCs likely influenced the degree of transmission heterogeneity observed during the study period.

Rather than estimating a conventional fixed dispersion parameter for each wave using aggregate per-wave data, we estimated a time-varying dispersion parameter. This is mainly because the data period setting for each wave is arbitrary when estimating fixed dispersion parameters, resulting in a biased estimation of dispersion parameters for different epidemic periods. Also, it was not possible to clearly define the epidemic period for each variant. In particular, the periods of the spread of Alpha and Delta variants are mixed. Furthermore, previous study has reported that non-time-varying estimates of dispersion parameters may overlook the possibility of large outbreaks^27^. In previous studies reporting time-varying dispersion parameters^23,27^, the values during the COVID-19 epidemic wave periods were continuously below one, consistent with the results of the present study.

Although substantial overdispersion was observed throughout the study period, these values fluctuated in the short term. Especially during two periods, in the latter half of the second wave (early April 2021) and the beginning of the fifth wave (January 2022), ${k}_{t}$ value exceeded one, suggesting that it was not highly overdispersed. Although the predominant variants (both D614G and Alpha were co-circulating in the second wave, while Omicron variants dominated in the fifth wave)^24,25^ and vaccination status differed between the two periods, they had one thing in common: a higher ${k}_{t}$ in the second wave was observed at the beginning of the fiscal year, with people’s movement associated with work and school transfers, and in the fifth wave was at the beginning of the new year, when many small gatherings were held, mainly as family gatherings. During these periods, there may have been many outbreaks among small groups of people rather than SSEs. Later, these small-group transmissions decreased, increasing the proportion of people who did not infect anyone; however, the SSEs were not eliminated. It can be seen from Fig. 1 that there were not many clusters during these periods, and that a short after, the number of clusters increased. Thus ${k}_{t}$ dropped sharply thereafter. This differs from the pattern of the Omicron epidemic exacerbated by large-scale party clusters, as reported in other countries^28–30^. Another possible reason for the high ${k}_{t}$ at the beginning of the fifth wave is the influence of ascertainment bias. Individuals who do not transmit the disease may be more likely to be missed by surveillance, especially when the number of cases is small^31^. In December 2021, when there were very few cases of COVID-19 in Yamagata Prefecture, it is possible that cases who did not infect anyone were more likely to be missed, resulting in an overestimation of ${k}_{t}$. In contrast, during the vacation period in mid-August 2021, known as the Obon period in Japan, the reproduction number was high (≥ 1), and the dispersion parameter was low (< 1), suggesting that many SSEs were occurring rather than a small group chain of transmission.

In this study, we showed the $R$ and $k$ separately for the transmission generations (G1 and G2+). It is clear from Fig. 3 that the reason $k$ is lower in G2+ than in G1 is not that SSEs are more likely to occur in G2+; rather, it is because most G2+ cases did not generate secondary cases. To our knowledge, only one study has estimated $k$ of COVID-19 separately for each generation showing that the value of $k$ became smaller as the generation proceeded^32^, which was consistent with our results. Given that $k$ is used in transmission models to quantify the uncertainty surrounding the estimation of the reproduction number and subsequent estimates of future case numbers^33,34^, we believe that it is important to estimate the parameter at least separately for G1 and G2+.

Our analysis was based on detailed forward and backward contact tracing conducted in Yamagata Prefecture. It has been shown mathematically using a branching process model that clusters can be found efficiently through backward contact tracing, especially in the presence of overdispersion^35^. Therefore, our results suggest that backward contact tracing is still beneficial after the emergence of VOCs and widespread vaccination. However, it is important to note that backward contact tracing, which investigates the source of infection, is logistically more burdensome than forward-tracing, which follows the people who have close contact with the case. When the number of cases increases significantly, it may not be feasible to investigate the sources of all cases. In such cases, because not all SSEs can be found and controlled, it would be effective to enforce measures such as limiting the number of people in restaurants and bars or reducing the number of large gatherings intensively to reduce random contacts with people other than those they meet regularly, depending on the epidemic situation^4^. Nevertheless, a practical, effective, and feasible system to obtain epidemiological parameters should be established. In addition, a continuous evaluation of the costs (workload and expenses) and effectiveness of such a system should be conducted.

Our study had some limitations. First, because we relied on public domain data, case ascertainment bias might have affected the results. In particular, after the Omicron variant emerged, the daily number of cases increased dramatically, posing a substantial burden on the public health agencies. This may have led to some cases being misclassified as having not caused secondary transmission. It must be noted that underreporting leads to an underestimation of *R*. It also leads to overestimation of *k* when SSEs are missed^36^. Second, because the sublineage BA.1^37^ of Omicron dominated during the study period, the overdispersion characteristics of other subvariants such as BA.2, BA,4, and BA.5, are unknown, and further study is warranted. Third, our analysis used data from only Yamagata Prefecture. Yamagata is a relatively small prefecture in terms of both population and population density, and the results may not generalize to Japan as a whole, or to other countries.

In conclusion, we showed substantial transmission heterogeneity throughout the epidemic period, predominated by VOCs (Alpha, Delta, and Omicron). We believe that it is important to continuously measure transmission dynamics in each region and implement appropriate countermeasures. Establishing a feasible system to obtain epidemiological information is vital to making this possible, and a continuous evaluation of its cost-effectiveness, depending on the epidemic situation, is also warranted for public health implications.

## Methods

### Ethical approval

The study was approved by the ethics committee of the Yamagata Prefectural Institute of Public Health (approval no. YPIPHEC 21-05). We used only anonymized publicly available data; therefore, the individual informed consent was waived. All methods were performed in accordance with the relevant guidelines and regulations.

### Setting

Yamagata Prefecture is in the northern part of Japan (Supplementary Fig. S4), with a population of 1.08 million. Among them, 50.5% are 20–64 years old, while 33.6% are 65 years old or over^38^. The population density ranks 44th (/47 prefectures) in Japan at 370.9 residents per square kilometer^39^. Between March 22 and April 25, 2021, a state of emergency was declared only in Yamagata City, the prefectural capital, and refraining from unnecessary outings and travel was recommended. In other periods, measures such as restrictions on gatherings and reduced restaurant opening hours were implemented depending on the epidemic situation. Because the impact of emergency declarations and other measures varies from place to place within a prefecture, we did not analyze the relationship between these intervention policies and the dispersion parameter. Using open data from the Digital Agency^40^, the percentage of vaccination coverage in the Yamagata Prefecture is shown in Supplementary Fig. S6. Almost all vaccines used in Japan were mRNA vaccines (Pfizer and Moderna). For those aged 65 years and older, the coverage of the second dose of vaccines reached 75% in late July 2021 and was approximately 94% as of January 1, 2022, while for younger people, the coverage was less than 70% as of January 1, 2022. The coverage of third-dose vaccination for all age groups during the study period was very low since booster vaccination started in early January 2022.

### COVID-19 case data and cluster data collection

Public health centers have been conducting backward contact tracing for COVID-19 cases to identify infection sources and transmission routes, in addition to active case findings from close contacts since the beginning of the epidemic in Yamagata^41^. The information collected by contact tracing has been put on prefectural and municipal websites to share the information with the general public^21^. We collected and collated these data, such as date of onset, confirmed date, and the source case or place of infection (if available), for analysis^22^. We limited the study period of the main analysis to January 19, 2022, by defining the cutoff as the upper limit of the confidence interval for the daily percentage of cases with an identified source of infection greater than 50% (Supplementary Fig. 1A). Aside from the information on individual cases, information on clusters (date of report, cluster size, and cluster type) was obtained from the Yamagata Prefecture website^42^.

### Observed offspring distribution

The definition of transmission pairs was based on a previous study^8^. We counted the number of secondary cases of each identified case based on the transmission pairs separately by the generation of transmission. Cases with an unknown source of infection or the earliest onset date among the identified transmission chains were regarded as G1, and the other cases were regarded as G2+. However, because there was a limitation in identifying the transmission pairs within the cluster cases after G2, clusters comprising several cases with primary exposure reported at a common event or venue were excluded from calculating the number of secondary cases of G2+. In addition, as a sensitivity analysis, we created a dataset of the number of secondary cases after excluding all cases associated with healthcare and facilities for the old and handicapped. This is because the clusters were generally larger, and multiple transmission generations might have occurred in these facilities, which may overestimate the transmission dispersion from a single primary case. Finally, we generated the offspring distribution based on the number of secondary cases from a single case each time.

### Estimating transmission heterogeneity and the proportion of cases infecting 80%

We fitted a negative binomial distribution to the observed offspring distribution by the Bayesian approach. The two parameters of the distribution, mean ($R$) and dispersion parameters ($k$), were estimated using the Hamiltonian Monte Carlo method (HMC). To estimate the time-varying dispersion parameter (${k}_{t}$), we subset cases in a fixed window that included cases from one week before and after the corresponding date. We check the validity of the window length by comparing the observed mean number of secondary cases with those estimated by the ${R}_{t}$ and ${k}_{t}$ posterior distribution (as detailed in the Supplementary Material). In estimating ${k}_{t}$, it sometimes diverged because the sample size in a given time window was too small to permit the estimation. Because of the instability of the estimation, those time windows were excluded from the results.

Assuming that the number of secondary cases ($X$) follows a negative binomial distribution with the estimated parameters ($R$ and $k$), the expected proportion of cases responsible for 80% (${P}_{80}$) of transmission was given by the following equation proposed by Endo et al.^1^.

$$1 - P_{80} = \int_{0}^{X} {NB\left( {\left\lfloor x \right\rfloor ;k,\frac{k}{R + k}} \right)} dx$$

where $$X$$ satisfies

$$0.2 = \frac{1}{R}\int_{0}^{X} {\left\lfloor x \right\rfloor NB\left( {\left\lfloor x \right\rfloor ;k,\frac{k}{R + k}} \right)} dx$$

The calculation can be eased by the following.

$$\frac{1}{R}\int_{0}^{X} {\left\lfloor x \right\rfloor NB\left( {\left\lfloor x \right\rfloor ;k,\frac{k}{R + k}} \right)} dx = \int_{0}^{X - 1} {NB\left( {\left\lfloor x \right\rfloor ;k + 1,\frac{k}{R + k}} \right)} dx$$

### Supplementary Information

Supplementary Information.

## Data Availability

The aggregate data and code are available by emailing the corresponding author upon reasonable request.

## References

[1] Endo A, Abbott S, Kucharski AJ, Funk S (2020). Estimating the overdispersion in COVID-19 transmission using outbreak sizes outside China. Wellcome Open Res..

[2] Adam DC (2020). Clustering and superspreading potential of SARS-CoV-2 infections in Hong Kong. Nat. Med..

[3] Lloyd-Smith JO, Schreiber SJ, Kopp PE, Getz WM (2005). Superspreading and the effect of individual variation on disease emergence. Nature.

[4] Sneppen K, Nielsen BF, Taylor RJ, Simonsen L (2021). Overdispersion in COVID-19 increases the effectiveness of limiting nonrepetitive contacts for transmission control. Proc. Natl. Acad. Sci. U. S. A..

[5] Kain MP, Childs ML, Becker AD, Mordecai EA (2021). Chopping the tail: How preventing superspreading can help to maintain COVID-19 control. Epidemics.

[6] Althouse BM (2020). Superspreading events in the transmission dynamics of SARS-CoV-2: Opportunities for interventions and control. PLoS Biol..

[7] Oshitani, H. Cluster-based approach to Coronavirus Disease 2019 (COVID-19) response in Japan—February–April 2020. *Jpn. J. Infect. Dis.* **73**, 491-493 (2020). 10.7883/yoken.JJID.2020.363 32611985

[8] Ko YK (2022). Secondary transmission of SARS-CoV-2 during the first two waves in Japan: Demographic characteristics and overdispersion. Int. J. Infect. Dis..

[9] Ogata, T. *et al.* Increased Secondary Attack Rates among the Household Contacts of Patients with the Omicron Variant of the Coronavirus Disease 2019 in Japan. *Int. J. Environ. Res. Public Health* **19**, 8068 (2022). 10.3390/ijerph19138068 PMC9266248 35805724

[10] Jørgensen SB, Nygård K, Kacelnik O, Telle K (2022). Secondary attack rates for omicron and delta variants of SARS-CoV-2 in Norwegian Households. JAMA J. Am. Med. Assoc..

[11] Ryu, S., Kim, D., Lim, J.-S., Ali, S. T. & Cowling, B. Serial Interval and Transmission Dynamics during SARS-CoV-2 Delta Variant Predominance South Korea. *Emerg. Infect. Dis. J.* **28**, 350 (2022). 10.3201/eid2802.211774 PMC8798673 34906289

[12] Hwang H (2022). Transmission Dynamics of the Delta Variant of SARS-CoV-2 Infections in South Korea. J. Infect. Dis..

[13] Zhao, S., Guo, Z., Chong, M. K. C., He, D. & Wang, M. H. (2022) Superspreading potential of SARS-CoV-2 Delta variants under intensive disease control measures in China. *J. Travel Med.* 1:2. doi: 10.1093/jtm/taac025. 10.1093/jtm/taac025 PMC8903516 35238919

[14] Mikszewski A, Stabile L, Buonanno G, Morawska L (2022). Increased close proximity airborne transmission of the SARS-CoV-2 Delta variant. Sci. Total Environ..

[15] Petros BA (2022). Early introduction and rise of the omicron severe acute respiratory syndrome coronavirus 2 (SARS-CoV-2) variant in highly vaccinated university populations. Clin. Infect. Dis..

[16] Guo Z (2022). Superspreading potential of COVID-19 outbreak seeded by Omicron variants of SARS-CoV-2 in Hong Kong. J. Travel. Med..

[17] Guo Z (2022). Superspreading potential of infection seeded by the SARS-CoV-2 Omicron BA.1 variant in South Korea. J. Infect..

[18] Wang J (2021). Superspreading and heterogeneity in transmission of SARS, MERS, and COVID-19: A systematic review. Comput. Struct. Biotechnol. J..

[19] Lewis D (2020). Why many countries failed at COVID contact-tracing-but some got it right. Nature.

[20] Kazuaki, J., Mieko, K.-C. & Hitoshi, O. *Response COVID-19*. https://responsecovid19.org/japan/data/.

[21] *New Coronavirus Infectious Disease Portal Site, Yamagata, Japan*. https://www-pref-yamagata-jp.translate.goog/090016/bosai/kochibou/kikikanri/covid19/shingata_corona.html?_x_tr_sl=ja&_x_tr_tl=en&_x_tr_hl=en.

[22] Ninomiya K, Kanamori M, Ikeda N, Jindai K (2022). Integration of publicly available case-based data for real-time coronavirus disease 2019 risk assessment, Japan. West. Pacific Surveill. Response J. WPSAR.

[23] Adam, D. *et al.* (2022). Time-varying transmission heterogeneity of SARS and COVID-19 in Hong Kong. *Reseach Sq.* 1:17

[24] Yusuke K (2022). Replacement of SARS-CoV-2 strains with variants carrying N501Y and L452R mutations in Japan: An epidemiological surveillance assessment. West. Pacific Surveill. Response J. WPSAR.

[25] *SARS-CoV-2 Genomic Surveillance (National Institute of Infectious Diseases)*. https://www.niid.go.jp/niid/images/cepr/covid-19/20220914_genome_surveillance.pdf.

[26] Hall V (2022). Protection against SARS-CoV-2 after Covid-19 Vaccination and Previous Infection. N. Engl. J. Med..

[27] Guo Z (2023). A statistical framework for tracking the time-varying superspreading potential of COVID-19 epidemic. Epidemics.

[28] Brandal LT (2021). Outbreak caused by the SARS-CoV-2 Omicron variant in Norway, November to December 2021. Eurosurveillance.

[29] Espenhain L (2021). Epidemiological characterisation of the first 785 SARS-CoV-2 Omicron variant cases in Denmark, December 2021. Eurosurveillance.

[30] NSW Health. *Public Health Alert: Sydney Harbour Boat Cruise*. https://www.health.nsw.gov.au/news/Pages/20211207_01.aspx (2021).

[31] Lloyd-Smith JO (2007). Maximum likelihood estimation of the negative binomial dispersion parameter for highly overdispersed data, with applications to infectious diseases. PLoS ONE.

[32] Shi Q (2021). Effective control of SARS-CoV-2 transmission in Wanzhou, China. Nat. Med..

[33] Linton NM, Akhmetzhanov AR, Nishiura H (2021). Localized end-of-outbreak determination for coronavirus disease 2019 (COVID-19): Examples from clusters in Japan. Int. J. Infect. Dis..

[34] Johnson KD (2021). Disease momentum: Estimating the reproduction number in the presence of superspreading. Infect. Dis. Model..

[35] Endo A (2021). Implication of backward contact tracing in the presence of overdispersed transmission in COVID-19 outbreaks [version 1; peer review: 2 Approved] Centre for the Mathematical Modelling of Infectious Diseases COVID-19 Working. Wellcome Open Res..

[36] Blumberg S, Lloyd-Smith JO (2013). Inference of R0 and transmission heterogeneity from the size distribution of stuttering chains. PLoS Comput. Biol..

[37] Ministry of Health, Labour and Welfare. *The 88th Meeting of the Advisory Board for COVID-19*. https://www.mhlw.go.jp/content/10900000/000955794.pdf (2022) .

[38] Ministry of Internal Affairs and Communications. *Population and Households in Japan Derived from the Basic Resident Registration (by Municipality)*. https://www.soumu.go.jp/main_sosiki/jichi_gyousei/daityo/jinkou_jinkoudoutai-setaisuu.html.

[39] e-Stat, Portal Site of Official Statistics of Japan. *System of Social and Demographic Statistics, Statistical Observations of Municipalities 2021, Natural Environment*. https://www.e-stat.go.jp/en/stat-search/files?page=1&layout=datalist&toukei=00200502&tstat=000001154566&cycle=0&tclass1=000001154567&tclass2val=0.

[40] Digital Agency. *Vaccination Record System (VRS)*. https://info.vrs.digital.go.jp/.

[41] Seto J (2021). Epidemiology of Coronavirus Disease 2019 in Yamagata Prefecture, Japan, January–May 2020: The importance of retrospective contact tracing. Jpn. J. Infect. Dis..

[42] *Group (Cluster) Outbreak Situation of New Coronavirus Infectious Disease*. https://www-pref-yamagata-jp.translate.goog/090016/kenfuku/kansensyou/covid19-outbreak.html?_x_tr_sl=ja&_x_tr_tl=en&_x_tr_hl=en.

