# Supplementary material for: Time-varying overdispersion of SARS-CoV-2 transmission during the periods when different variants of concern were circulating in Japan
Source: Sci Rep. 2023 Aug 14;13:13230. doi: 10.1038/s41598-023-38007-x (PMC10425347; doi:10.1038/s41598-023-38007-x)
Supplement: Supplementary file 1 — Supplementary Information. [file 41598_2023_38007_MOESM1_ESM.docx]

**Title:** Time-varying overdispersion of SARS-CoV-2 transmission during the periods when different variants of concern were circulating in Japan

Contents

[1. Supplementary Methods and Results 1](#_Toc116595512)

[2. Figures 4](#_Toc116595513)

[3. Tables 10](#_Toc116595514)

[4. References 11](#_Toc116595515)

#

# **1. Supplementary Methods and Results**

*Fitting a negative binomial distribution to the observed offspring distribution*

We employed a Bayesian framework to estimate the parameters ($R_{t}$, $k_{t}$). Both parameters are estimated using uniform $U(0,10000)$ priors. We employed Hamiltonian Monte Carlo method (HMC) to sample the posterior distribution, using four independent chains of 10,000 samples, of which 1,000 were retained post-thinning; the first 1,000 steps of each chain were discarded as a warm-up. The HMC method was implemented using Stan [1], specifically the cmdstan [2] package (version 2.29.2) in R version 4.0.3 [3].

We estimated the posterior distribution for each time point individually, using a sliding window of data around the time point of length $W$. Setting $W$ to be small would lead to estimates that have low precision due to the paucity of data within the window, while setting it to be large would lead to overly smooth estimates that are not responsive to localized changes. We therefore sought to validate the choice of $W$ using the coverage of the derived prediction intervals as described below.

*Validation of the length of the sliding window*

We assessed the validity of the window length by the procedure outlined below, which uses the following notation.

| $R_{st}$ | The $s$th sample from the posterior for the reproduction number derived from Stan applied to the sliding window around time $t;$ |
| --- | --- |
| $K_{st}$ | The $s$th sample from the posterior for the overdispersion factor, also around time $t$; |
| $x_{it}$ | The $i$th sample number of secondary cases from a cluster whose primary case had onset on day $t$; |
| $n_{t}$ | Number of data samples on day $t$; and |
| $\tilde{x}_{sit}$ | Simulated data sample $i$ from day $t$ using the $s$th sample from the posterior. |

The following steps were conducted:

1. We draw samples $R_{st}$ and $K_{st}$ by fitting a negative binomial model to the observed offspring distribution using the method described above for each sliding window of length $W$ (suppressed from notation for simplicity) around time $t$.

2. We then simulate $\tilde{x}_{sit}$ for each cluster $i$ with onset on day $t$ from a negative binomial model with parameters $R_{st}$ and $K_{st}$.

3. From this, we calculate the simulated mean $\bar{\chi}_{st}=\sum_{i=1}^{n_{t}} \tilde{x}_{sit}/n_{t}$.

4. We then calculate an equal-tailed 95% prediction interval from the quantiles of $\bar{\chi}_{st}$.

5. We then check if the data mean $\bar{x}_{t}=\sum_{i=1}^{n_{t}} x_{it}/n_{t}$ is inside the interval or not.

By implementing the procedure for each transmission generation for $W=15$, we found that the proportion of time that the data mean $\bar{x}_{t}$ is inside the interval were 97.1%, 98.2%, and 96.5% for G1, G2+, and all generations, respectively (Supplementary Figure S4). Thus, we regarded the sliding window length as appropriate for this dataset.

# **2. Figures**

**
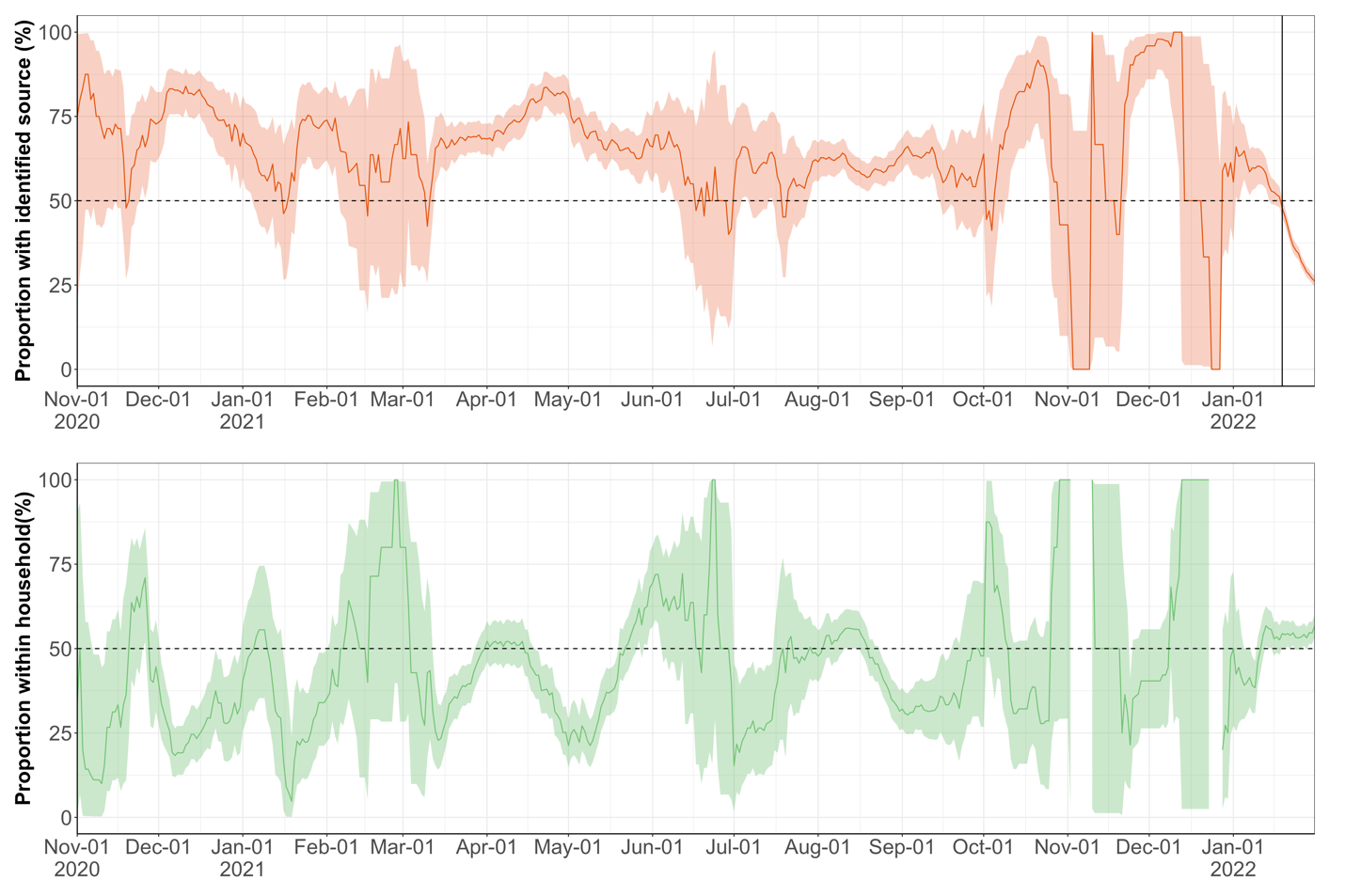
Supplementary Figure S1.** Weekly proportion of A) cases with an identified source and B) cases infected within a household (15 days moving average). The shaded areas show the 95% CI.


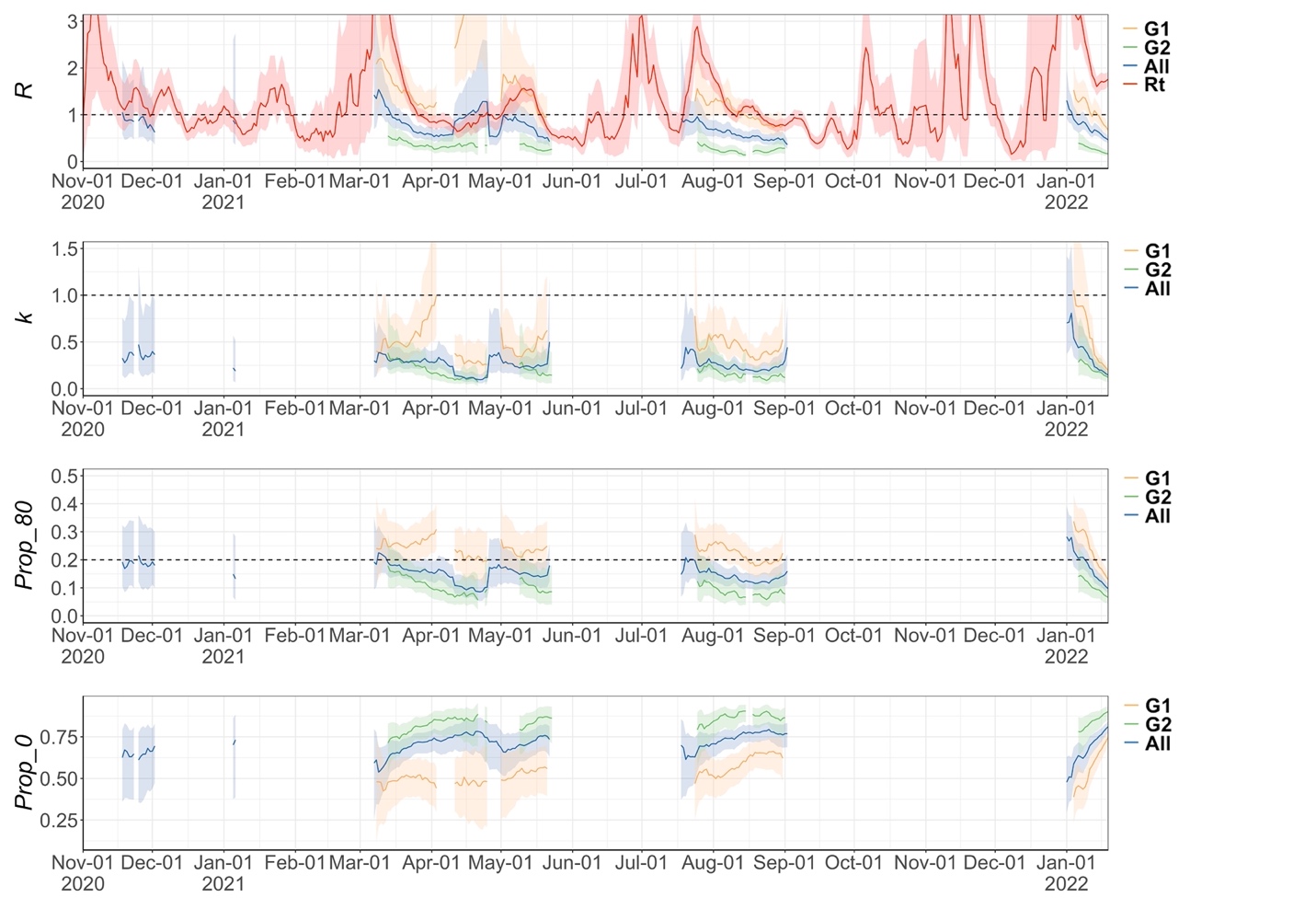


**Supplementary Figure S2.** A) Effective reproduction number ($R_{t}$, or *R* on the chart), red line was estimated from the daily number of cases, B) time-varying dispersion parameter ($k_{t}$, *k* on the chart), C) the proportion of cases infecting 80% ($P_{80}$, *Prop_80* on the chart), and D) the proportion of cases who did not spread to anyone ($P_{0}$, *Prop_0* on the chart) for each generation of transmission in Yamagata, Japan between 2020 November 1 and 2022 January 19. The shaded areas show the 95% CrI.

G1: first-generation, G2+: second-generation or later, All: All generation


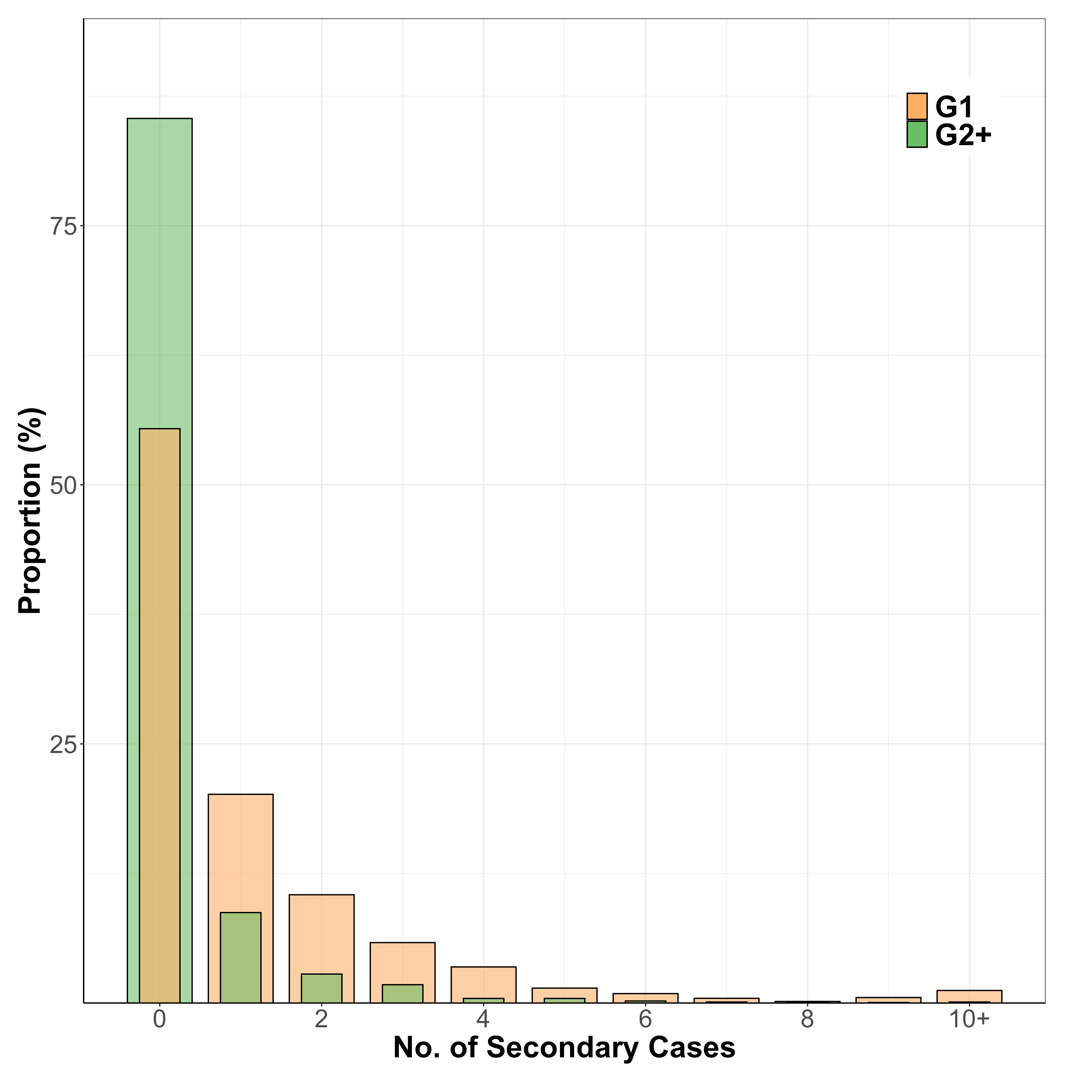


**Supplementary Figure S3.** The observed offspring distribution of the number of secondary cases from G1 and G2+ cases after excluding cases associated with healthcare and other facilities in Yamagata, Japan.

G1: first-generation, G2+: second-generation or later

**
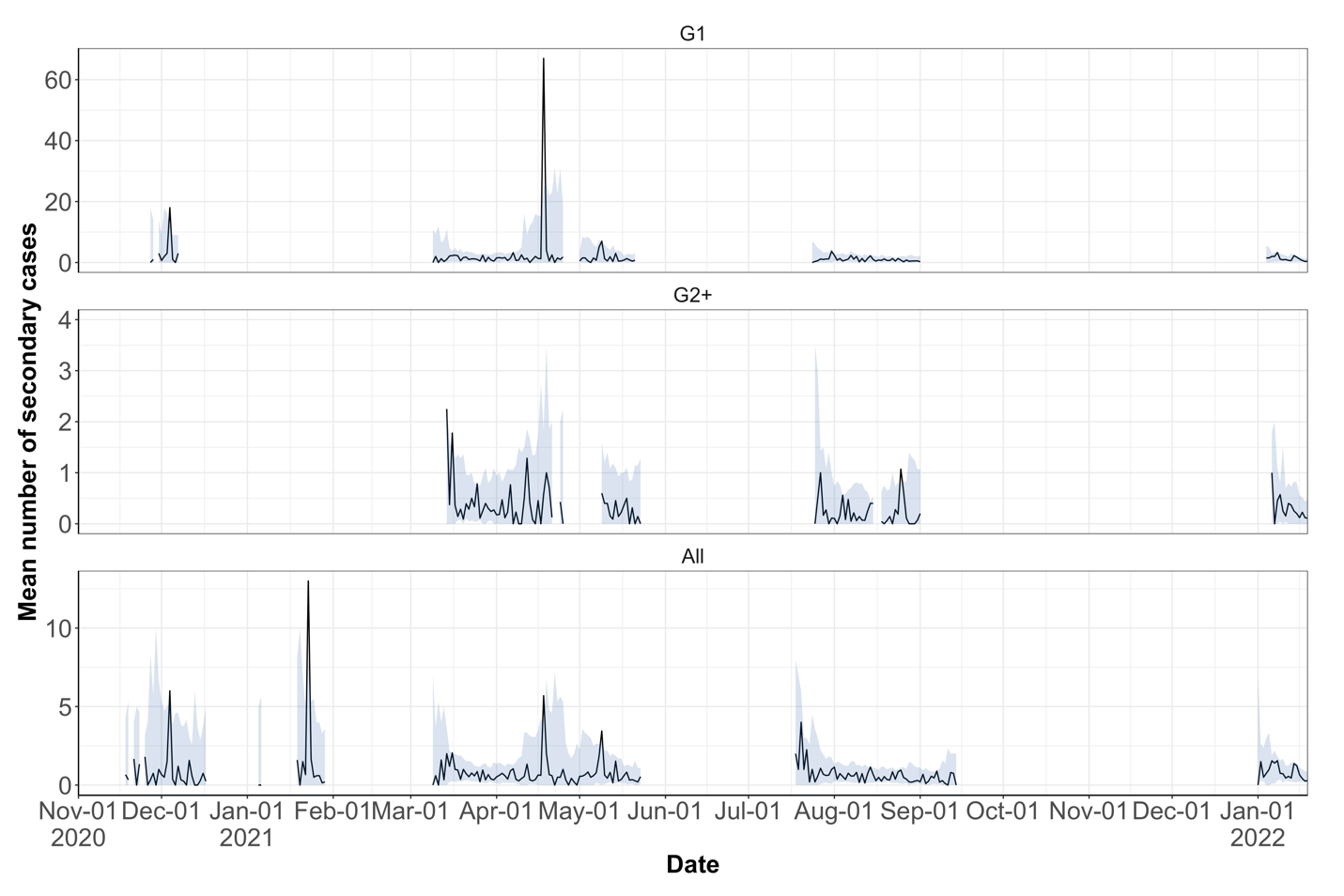
**

**Supplementary Figure S4.** The observed mean number of secondary cases (line) and the 95% prediction of the mean number of secondary cases estimated using the $R_{t}$ and $k_{t}$ posterior distribution (shading).

**
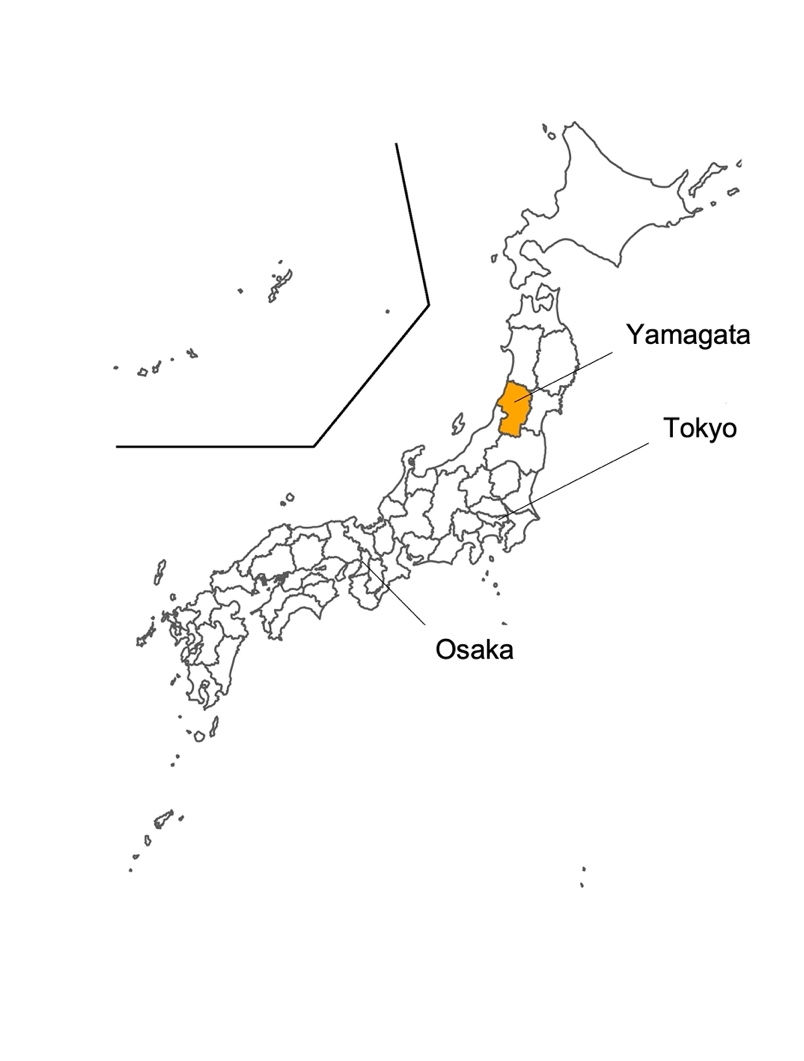
**

**Supplementary Figure S5.** Yamagata Prefecture Location. It is in the Tohoku region of Japan.


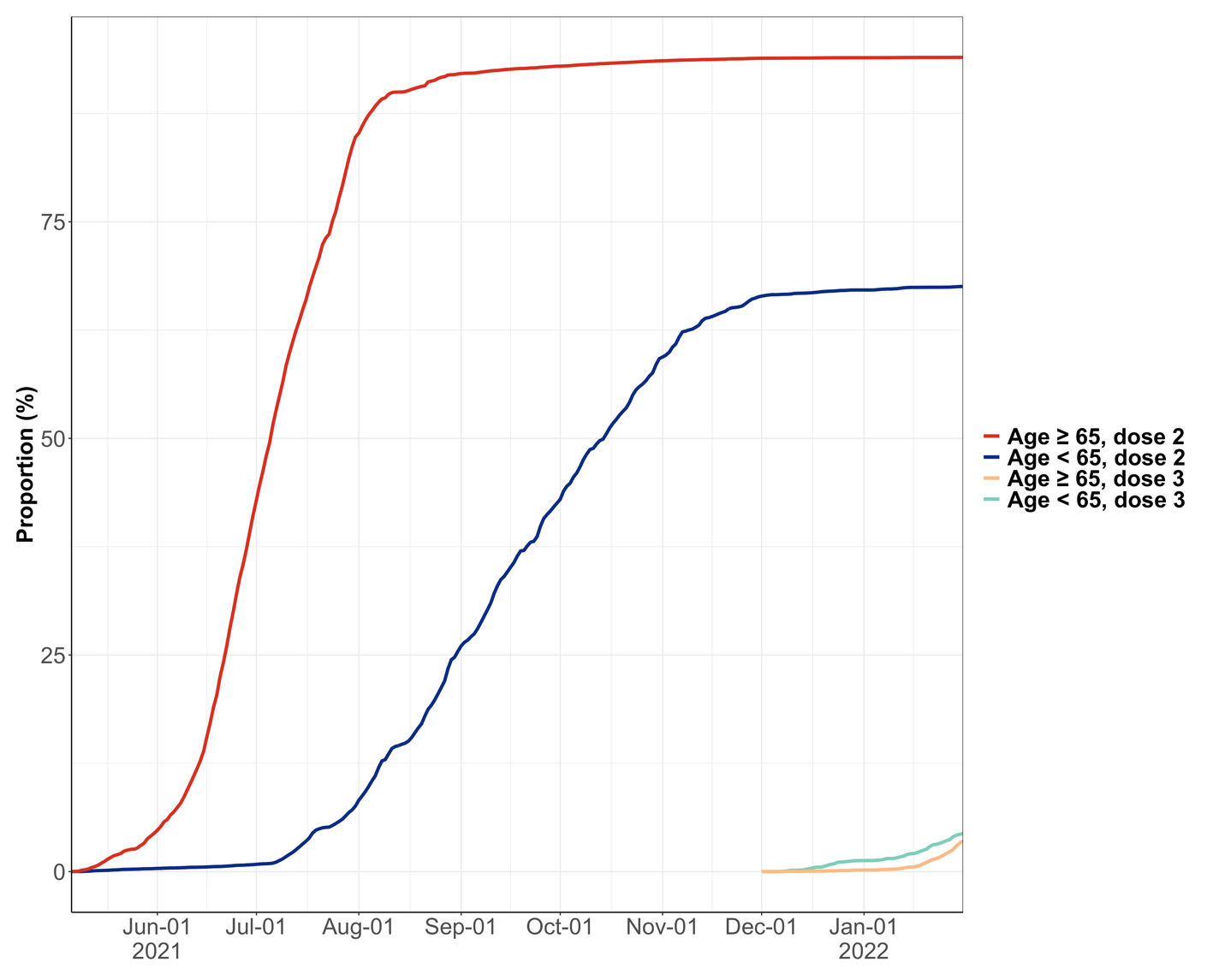


**Supplementary Figure S6.** The proportion of the population vaccinated with second and third dose by age group in Yamagata Prefecture.

# **3. Tables**

**Supplementary Table S1.** Estimated time-fixed reproduction number ($R$), dispersion parameter ($k$), the proportion of cases responsible for 80% of transmission ($P_{80}$), and the proportion of cases who did not spread to anyone ($P_{0}$) by generation of transmission after excluding cases associated with healthcare and other facilities.

| **Generation** | ***R*** | ***k*** | $\boldsymbol{P}_{\boldsymbol{80}}$ | $\boldsymbol{P}_{\boldsymbol{0}}$ |
| --- | --- | --- | --- | --- |
| G1 | 1.16 (1.05–1.28) | 0.45 (0.39–0.52) | 0.23 (0.21–0.25) | 0.56 (0.53–0.60) |
| G2+ | 0.28 (0.24–0.32) | 0.16 (0.13–0.19) | 0.09 (0.08–0.11) | 0.85 (0.83–0.87) |
| All | 0.62 (0.57–0.67) | 0.24 (0.21–0.26) | 0.14 (0.13–0.15) | 0.74 (0.72–0.76) |

# **4. References**

1. Stan Reference Manual Version 2.30 [Internet]. Available from: https://mc-stan.org/docs/reference-manual/index.html#overview

2. CmdStan, the shell interface to Stan [Internet]. Available from: https://mc-stan.org/users/interfaces/cmdstan

3. R Core Team (2021). R: A language and environment for statistical computing. R Foundation for Statistical Computing, Vienna, Austria [Internet]. Available from: https://www.r-project.org/
